# Population prevalence of *Chlamydia trachomatis *and *Neisseria gonorrhoeae *in the Netherlands. should asymptomatic persons be tested during Population-based chlamydia Screening also for gonorrhoea or only if chlamydial infection is found?

**DOI:** 10.1186/1471-2334-6-42

**Published:** 2006-03-07

**Authors:** Jan EAM van Bergen, Joke Spaargaren, Hannelore M Götz, Irene K Veldhuijzen, Patrick JE Bindels, Ton J Coenen, Jan Broer, Fetzen de Groot, Christian JPA Hoebe, Jan-Hendrik Richardus, Daniel van Schaik, Marije Verhooren, the PILOT CT study-group*

**Affiliations:** 1 STI AIDS Netherlands (Soa Aids Nederland), Amsterdam, The Netherlands; 2 Municipal Public Health Service Rotterdam, The Netherlands; 3 Municipal Public Health Service South Limburg, The Netherlands; 4 Municipal Public Health Service Groningen, The Netherlands; 5 Department of Public Health, Erasmus MC, University Medical Center Rotterdam, The Netherlands; 6 Department of General Practice, Academic Medical Centre-University of Amsterdam, The Netherlands; 7 Municipal Public Health Service "Hart van Brabant", The Netherlands; 8 Municipal Public Health Laboratory GGD Amsterdam, The Netherlands

## Abstract

**Background:**

Screening and active case finding for *Chlamydia trachomatis *(CT) is recommended to prevent reproductive morbidity. However insight in community prevalence of gonococcal infections and co-infections with *Neisseria gonorrhoea *(NG) is lacking.

**Methods:**

Nested study within a large population-based Chlamydia Screening Pilot among 21.000 persons 15–29 year. All CT-positive (166) and a random sample of 605 CT-negative specimens were as well tested for gonococcal infection.

**Results:**

Overall Chlamydia prevalence in the Pilot was 2.0% (95% CI: 1.7–2.3), highest in very urban settings (3.2%; 95% CI: 2.4–4.0) and dependent of several risk factors. Four gonococcal infections were found among 166 participants with CT infection (4/166 = 2.4%; 95% CI: 0.1%–4.7%). All four had several risk factors and reported symptoms. Among 605 CT-negative persons, no infection with NG could be confirmed.

**Conclusion:**

A low rate of co-infections and a very low community prevalence of gonococcal infections were found in this population based screening programme among young adults in the Netherlands. Population screening for asymptomatic gonococcal infections is not indicated in the Netherlands. Although co-infection with gonorrhoea among CT-positives is dependent on symptoms and well-known algorithms for elevated risks, we advise to test all CT-positives also for NG, whether symptomatic or asymptomatic.

## Background

Chlamydial and gonococcal infections are important causes of reproductive morbidity [1-3]. Nucleic Acid Amplication tests (NAATs) on self-obtained specimens (urine, vaginal swabs) make it feasible to detect these infections in a very effective manner, inside as well as outside conventional clinic settings [4-6]. In fact these new technologies prelude a potential revolution in our ability to control Sexually Transmitted Infections (STI). The vast majority of STI is asymptomatic or sub-clinical and these "hidden infections" are the key to persistence and ongoing transmission on a population level. Merely treatment of symptomatic cases will not be able to influence transmission dynamics significantly.

Therefore, in many countries screening or active case finding for *Chlamydia trachomatis *(CT) is recommended. Although information on population prevalence of CT becomes more widespread, unfortunately little information on population prevalence of gonococcal infections in the general young adult population is available. As the feasibility of combined testing increases, this lack of information hampers insight in the question whether or not to integrate testing for *Neisseria gonorrhoeae *(NG) in Chlamydia screening programmes. Insight in the rate of NG (co-)infections in asymptomatic persons could fuel cost effectivity analysis and offer evidence-based information about the need for persons found positive in Chlamydia screening to be tested for NG co-infection as well. Although patients at STI clinics get a full STI screen even if asymptomatic, this is not a routine procedure in primary care. In the Netherlands health care seeking behaviour for STI is geared towards primary care, the General Practitioner (GP) addressing the majority of the STI-related problems[7].

We wanted to estimate community prevalence of NG infections and the number of dual infections in CT infected participants in a population based screening programme in the Netherlands.

## Methods

A large population based Chlamydia screening was performed (2003) by inviting 21.000 persons in urban and rural areas for home-based urine testing. Design and results of this study has been described in detail elsewhere[8]. In summary, this representative cross-sectional study was a stratified national probability survey according to 'area address density'. 21000 random-selected women and men in 4 regions, aged 15–29 years, received a home-sampling kit and a questionnaire. Urine-samples were returned by mail, pooled by 5 and tested by polymerase chain reaction (PCR Roche Diagnostic Corp., Indianapolis, IN, USA). Positive pools were individually retested. Treatment was possible via the GP, STI- or MHS-clinic. 82% of patients that were tested positive in our home-based CT screening program went to the GP for treatment.

For the current research question all Chlamydia positive (n = 166) and a random sample of 605 Chlamydia negative urine specimens (out of a total 8217 negatives) were as well tested for NG infection according the manufacturer's instructions (Roche Diagnostic Corp., Indianapolis, IN, USA). Confirmation of NG positive results was performed by detecting the *cppB *gene and the multicopy *opa *genes with a real-time PCR method using the Rotorgene instrument[9,10].

## Results

In the initial Chlamydia Screening Study 10.610 persons responded: 11% sent in a refusal card and 41% (n = 8383) participated by sending in urine and questionnaire. Non-response analysis showed a balance of high and low risk categories among participants. Details have been reported elsewhere[8,11]. Overall Chlamydia prevalence was 2.0% (95% CI: 1.7–2.3); 2.5% (2.0–3.0%) in women and 1.5% (1.1–1.9) in men. Chlamydia prevalence was significantly higher in very high urbanised areas 3.2% (95% CI: 2.4–4.0) compared to rural areas 0.6% (0.1–1.1). Infection was also associated with self-reported ethnicity (especially Surinamese/Antillean 8.2% [95% CI: 3.9–12.5]), number of sex partners and symptoms.

Among 166 samples of persons who tested positive for *Chlamydia trachomatis *infection, 4 gonococcal infections were diagnosed (4/166 = 2.4%. 95% CI: 0.1%–4.7%). Initially 9/166 were reactive, but only 4 out of 9 were positive in confirmatory PCR NG testing. All 4 persons co-infected with NG were either 17 or 18 year, 3 reported 6–10 lifetime partners (and 2 had 2–5 partners in the past 6 month). All 4 reported symptoms (lower abdominal pain, intermenstrual bleeding, dysuria) and no condom-use during last sex contact. Two reported a Surinamese-Antillean background. Main characteristics and riskfactors of the 4 dually infected persons are listed in table 1. Among 605 Chlamydia-negative persons, no gonococcal infections were diagnosed. Initially 16/605 were reactive for NG, but none could be confirmed with the additional confirmatory test.

**Table 1:** Main characteristics and risk factors of the 4 dually infected CT-positive and NG-positive cases

	**Case 1**	**Case 2**	**Case 3**	**Case 4**
Sex	female	female	female	female
Age	17 yr	17 yr	17 yr	18 yr
Ethnicity	Dutch	Surinamese	Dutch	Surinamese
Level of urbanisation *	high urban	very high urban	very high urban	high urban
Level of education	unknown	intermediate	low	high
Symptoms:				
Intermenstrual and/or postcoital bleeding	+	-	+	-
Painfull/frequent mixturation	+	+	+	+
Lower abdominal pain	-	+	+	+
Lifetime partners	2–5	6–10	6–10	6–10
Partners previous 6 months	1	1	2–5	2–5
New partner previous 2 months	-	-	+	+
Condom use last sexual contact	-	-	-	-
History of previous STI	+	-	+	-

* According to Area Address Density: very high urban (> 2500 addresses/km^2^); high urban (1500–2500 addresses/km^2^); moderate urban (1000–1500 addresses/km^2^);) low urban (500–1000 addresses/km^2^); and rural (<500 addresses/km^2^) .

## Conclusion

In this large national representative population based Chlamydia Screening in the Netherlands among 21.000 persons we have reported an overall CT prevalence of 2.0 %. In order to gain insight in NG dual infections we retested all positive CT specimens and found a low rate of NG co-infections (2.4%; 4/166) among CT positives. We found no NG infections at all among a random subset of 605 CT negative samples, suggesting a very low community prevalence of gonococcal infections in the young adult population. Given this very low NG prevalence, general population screening for asymptomatic NG infections is not indicated in the Netherlands and targeted screening is a better and more cost-effective option. For instance, at the Amsterdam STI clinic 10% of the (heterosexual) visitors had CT infection and 2.5% GC infection; with much higher rates in MSM (CT:13% NG:14%) and in Surinamese-Antillean population (CT: 16% NG: 7.6%)[12]. The inequalities in rates of CT and NG in black ethnic groups are well known[13,14]. We also found considerable higher CT prevalence (8.2%) among Surinamese Antillean persons in our initial CT screening, and ethnicity remained an independent variable in our prediction rule for selective CT-screening[11]. Also 2 out of the 4 positive NG participants had a Surinamese/Antillean background, which is remarkable because only 1.6% of all participants in our screening belonged to this ethnicity. NG infections are even more than CT infections concentrated within particular risk groups, within specific risk networks and entangled in specific risk environments[15].

The number of reported dual infections in the literature varies considerably, from less than 1% up to more than 40% [16-18]. Most studies have been performed in clinical settings among selected patient groups and often relate to the proportion concurrent CT infections in NG infected persons. This relation has been reported consistently high and justifies the policy of giving antibiotic treatment for Chlamydia at the time of Gonorrhoea diagnoses, when CT results are not available. However, the opposite -concurrent infection with NG if CT is diagnosed – is less often the case, and even less in CT cases found in home- or community-based screening programmes. In a Chlamydia screening programme in the UK, prevalence of gonorrhoea among CT-positives was 4.6% for women and 6.3% for men in STI clinics but only 0.2% for women and 1.2% for men in the CT-positives found via community screening[19]. In the US, a nationally representative prevalence study, found a CT prevalence of 4.2% and a low infection rate for NG (0.43%) and prevalence of co-infection was only 0.3%[20]. Substantial racial/ethnic disparities in prevalence of both infections were reported. Some regional home surveys in the US reported substantially higher NG prevalence (5.3% from the Baltimore's household survey, and 3.9% in San Francisco)[5].

We could not confirm the majority of our initially positive NG results as true positives. Certain strains of *Neisseriaceae*, considered as commensal organisms and *Lactobaccillus *species are known to produce false-positive results. This underlines once more the necessity of confirmatory testing in a screening programme, with a test that is more specific and at least as sensitive[9,10].

The few persons testing positive for NG in our study were all young women (17, 18 year) with a high risk profile (> 6 lifetime partners, no condom use during last sex and two had Surinamese/Antillean ethnicity). All reported in the questionnaire subjective complaints. This means that these patients, who came to the doctor for their treatment for the CT infection detected by home-based screening, are in fact entitled for a STI screen according to current guidelines and algorithms (symptomatic patients with a risk profile should be tested both for CT and NG). This suggests that even in participants who turn out CT-positive in a population screening programme in a low prevalence area, a routine NG screen would not be required if proper risk-assesment is made by the physician to tailor further need for a full STI screen. However, risk assessment in primary care is not always optimal and discussing sexual health in GP is not always easy, not for the doctor, nor for the patient[21]. We would argue therefore that pursuing in primary care the old paradigm: "always look for another STI if one STD is found" would be most practical. However, cost-effectiveness of such a strategy would depend very much on regional STI epidemiology.

As integrated combo-tests for diagnosing CT, NG, but also for Trichomonas, Mycoplasma, and even HIV might become within reach in the near future, and incremental costs for testing for these additional STI will become more favourable from a cost-effective point of view, special consideration should be paid to the potential negative side-effects and the enhanced likelihood of false-positive results if screening takes place in very low prevalence settings[22].

## Conclusion

Based on our results, population screening for gonococcal infections is not indicated in the Netherlands. NG co-infection in persons who tested Chlamydia positive in population screening programmes in young adults is dependent on symptoms and well known algorithms for elevated risks. Routine screening for dual infections in CT-positive participants is still recommended, whether symptomatic or asymptomatic. Compared to other countries, the Netherlands still has a low burden of STI.

## Competing interests

The author(s) declare that they have no competing interests.

## Authors' contributions

J.E.A.M. van Bergen was project leader, involved in conception, design and organisation of PILOT CT, interpretation of data and writing the first draft of the report. J. Spaargaren was responsible for analysis of the laboratory data and assisted in the first and final draft. H.M. Götz, I.K. Veldhuijzen, J.H. Richardus, C.J.P.A. Hoebe, J. Broer, A.J.J. Coenen, F. de Groot, D.T van Schaik, and M.J.C. Verhooren were all involved in conception, design, organisation and interpretation of the data. P.J.E. Bindels was in involved in conceptualising this sub-analysis, critically reviewed the first draft and assisted in the final report.

## Pre-publication history

The pre-publication history for this paper can be accessed here:


